# Identification of Putative β-Galactosidase Genes in the Genome of *Lactobacillus helveticus* OSU-PECh-4A

**DOI:** 10.1128/MRA.00766-21

**Published:** 2022-01-06

**Authors:** Israel García-Cano, Alejandra Escobar-Zepeda, Silvette Ruiz-Ramírez, Diana Rocha-Mendoza, Rafael Jiménez-Flores

**Affiliations:** a Department of Food Science and Technology, The Ohio State University, Columbus, Ohio, USA; b Host-Microbiota Interactions Laboratory, Wellcome Sanger Institute, Wellcome Genome Campus, Hinxton, United Kingdom; c Microbiome Informatics Team, European Bioinformatics Institute, Wellcome Genome Campus, Hinxton, United Kingdom; Indiana University, Bloomington

## Abstract

The Lactobacillus helveticus OSU-PECh-4A strain, from the Ohio State University Parker Chair collection, produces exceptional β-galactosidase activity using acid whey as a culture medium, compared with a commercial broth. The strain has a genome sequence of 1,834,843 bp, and its GC content is 36.69%. Using InterProScan v5.50-84.0 software, four genes with putative β-galactosidase function were found.

## ANNOUNCEMENT

Lactobacillus helveticus strain OSU-PECh-4A was isolated from commercial fermented milk (Columbus, OH, USA). Twenty-five grams of sample was mixed with 225 mL of sterile saline solution (0.85% NaCl [pH 7.0]). Serial dilutions were performed and plated on MRS agar (BD Difco, USA). The plates were incubated under aerobic conditions for 16 h at 37°C. The colonies were selected based on phenotypic features, i.e., shape, color, and texture. Using a synthetic substrate, the OSU-PECh-4A strain showed 5 times more β-galactosidase activity when it was cultivated in acid whey (AW) as a medium, compared with the commercial broth (MRS broth). The relative expression of the *bgal-*620 gene was 3 times higher in AW than in the MRS medium (1). It has been reported that lactic acid bacteria (LAB) have two overlapping genes (*lacL* and *lacM*) for β-galactosidase production (2). However, LAB can contain one, two, or three genes for the production of β-galactosidase in their genomes. The draft genome sequence of this strain should facilitate the identification of the putative genes encoding prospective β-galactosidase proteins and the understanding of the high activity levels shown by L. helveticus OSU-PECh-4A.

For genomic DNA (gDNA) extraction from L. helveticus OSU-PECh-4A, a purification kit (Wizard gDNA kit; Promega, USA) was used. Previously, the cells were grown in MRS broth (BD Difco, USA) and recovered by centrifugation at 10,000 × *g* for 10 min. The concentration and quality of the gDNA were measured using the PicoGreen method (catalog number P7589; Life Technologies, USA) and a 2200 TapeStation system (Agilent Technologies, Inc., USA), respectively. The DNA concentration used for the sequencing step was ∼50 ng/μL, with a DNA Integrity Number (DIN) value (with the Agilent 2200 TapeStation system and the Agilent gDNA ScreenTape assay) of 9.7.

The gDNA was used for Illumina high-throughput sequencing (NovaSeq 6000 S4 system; Illumina). The library was constructed following the TruSeq DNA PCR-free protocol, and 151 cycles of paired-end sequencing were performed at Psomagen (Rockville, MD, USA). A total of 14.28 million raw reads were processed. Default parameters were used except where otherwise noted. For quality control, we used Fastp v1.14.5 software (3); 98.88% of reads passed quality control and were used for genomic assembly with the SPAdes genome assembler v3.15 in mode --careful (4). We filtered out fragments shorter than 500 bp and computed the assembly statistics using in-house-built scripts (available at https://github.com/Ales-ibt/in_house_scripts). The L. helveticus OSU-PECh-4A genome is fragmented in 146 contigs (*N*_50_, 20,442 bp; *L*_50_, 27; *N*_90_, 6,481 bp; *L*_90_, 84), likely due to the presence of many repetitive sequences according to the large number of transposases encountered (130 genes). The genome size is 1,834,843 bp, and the GC content is 36.69%. According to CheckM v1.1.2 (5), this genome has 99.03% completeness and 0.00% contamination. Taxonomic assignment to L. helveticus was corroborated using GTDB-Tk v1.5.0 (6). Additionally, we computed the average nucleotide identity (ANI) versus 21 complete genome assemblies of Lactobacillus helveticus strains from RefSeq using the FastANI tool v1.3 (7). This analysis revealed that the two closest reference strains are L. helveticus strain D76 (GenBank accession number CP016827.1) and L. helveticus isolate MGYG-HGUT-02384 (GenBank accession number LR698986.1), both with 99.96% ANI.

According to gene prediction and functional annotation by NCBI Prokaryotic Genome Annotation Pipeline (PGAP) v5.2, this assembly has 1,929 total genes, 2 copies of 16S rRNA, and 51 genes encoding tRNAs. Additional annotation of functional domains in the amino acid sequences retrieved by Prokka v1.14.5 (8) was performed using InterProScan v5.50-84.0 (9) for the identification of genes with putative β-galactosidase function (Table 1). Four genes with this putative function were found in the L. helveticus OSU-PECh-4A genome. Two genes are contiguous and represent the large and small β-galactosidase subunits. The other two genes encode different proteins. The gene sequences and the amino acid sequences for the four genes detected did not show similarity to each other, as observed by multiple sequence alignment using MUSCLE v3.32.0 (10).

**TABLE 1:** Identification of genes with putative β-galactosidase function in L. helveticus OSU-PECh-4A using Prokka and InterProScan software^*a*^

Prokka gene ID	Contig ID	Nucleotide position		Strand	Gene name	UniProtKB annot	IPS database annot	IPS database ID(s)	IPS annot
		Start	End						
NIPFOCJE_01570	NODE_62_length_11113_cov_1011.571856	187	2073	+	*lacL*	β-Galactosidase large subunit	Pfam	PF00703, PF02836, PF02837	Glycosyl hydrolase family 2; glycosyl hydrolase family 2, TIM barrel domain; glycosyl hydrolase family 2, sugar-binding domain
NIPFOCJE_01571	NODE_62_length_11113_cov_1011.571856	2057	3013	+	*lacM*	β-Galactosidase small subunit	Pfam	PF02929	β-Galactosidase small chain
NIPFOCJE_01818	NODE_88_length_5952_cov_940.937021	485	1558	−	*lacZ*	β-Galactosidase LacZ	Pfam	PF02449	β-Galactosidase
NIPFOCJE_01932	NODE_107_length_3404_cov_922.981671	1303	1884	−	*lacG*	6-Phospho-β-galactosidase	Pfam	PF00232	Glycosyl hydrolase family 1

a ID, identification; annot, annotation; IPS, InterProScan; TIM, triosephosphateisomerase.

### Data availability.

The Lactobacillus helveticus OSU-PECh-4A draft genome was deposited in the NCBI database under the BioProject and BioSample accessions numbers PRJNA746544 and SAMN20209453, respectively. The Sequence Read Archive (SRA) accession number is SRR15131330. The GenBank accession number for the whole-genome sequence is JAHWBM000000000, and the GenBank accession number for the 16S rRNA gene is MW810614.1.
